# Supplementary material for: High-altitude Hypoxia Influences the Activities of the Drug-Metabolizing Enzyme CYP3A1 and the Pharmacokinetics of Four Cardiovascular System Drugs
Source: Pharmaceuticals (Basel). 2022 Oct 21;15(10):1303. doi: 10.3390/ph15101303 (PMC9612038; doi:10.3390/ph15101303)
Supplement: Supplementary file 1 [file pharmaceuticals-15-01303-s001.zip › Table S1.pdf]

The qPCR raw data of CYP3A1

	CT (CYP3A1)	CT (β-actin)	ΔCT	ΔΔCT	-ΔΔCT	$2^{-\Delta\Delta CT}$	mean	SD
normoxia group	17.085	16.966	0.12	0.42	-0.42	0.75	1.013	0.182
	17.345	17.619	-0.27	0.03	-0.03	0.98		
	17.168	17.170	0.00	0.30	-0.30	0.81		
	16.101	16.434	-0.33	-0.03	0.03	1.02		
	16.570	16.624	-0.05	0.25	-0.25	0.84		
	17.239	17.661	-0.42	-0.12	0.12	1.09		
	15.420	15.829	-0.41	-0.11	0.11	1.08		
	15.896	16.664	-0.77	-0.47	0.47	1.38		
	16.458	16.911	-0.45	-0.15	0.15	1.11		
	16.613	16.995	-0.38	-0.08	0.08	1.06		
acute hypoxia group	19.163	17.409	1.75	2.05	-2.05	0.24	0.484	0.217
	16.959	17.004	-0.05	0.25	-0.25	0.84		
	17.695	16.871	0.82	1.12	-1.12	0.46		
	17.530	17.312	0.22	0.52	-0.52	0.70		
	17.385	16.054	1.33	1.63	-1.63	0.32		
	17.686	16.641	1.05	1.35	-1.35	0.39		
	17.657	16.646	1.01	1.31	-1.31	0.40		
	16.851	16.739	0.11	0.41	-0.41	0.75		
	17.562	16.941	0.62	0.92	-0.92	0.53		
	18.955	16.963	1.99	2.29	-2.29	0.20		
chronic hypoxia group	19.386	17.834	1.55	1.85	-1.85	0.28	0.357	0.094
	18.503	17.000	1.50	1.80	-1.80	0.29		
	18.734	17.065	1.67	1.97	-1.97	0.26		
	17.892	17.135	0.76	1.06	-1.06	0.48		
	17.961	17.036	0.92	1.22	-1.22	0.43		
	17.919	16.873	1.05	1.35	-1.35	0.39		
	18.273	17.370	0.90	1.20	-1.20	0.43		
	19.437	18.127	1.31	1.61	-1.61	0.33		
	19.413	17.552	1.86	2.16	-2.16	0.22		
	17.833	17.028	0.80	1.10	-1.10	0.47		
chronic hypoxia return to normoxia group	18.608	17.353	1.26	1.56	-1.56	0.34	0.430	0.132
	18.116	17.540	0.58	0.88	-0.88	0.54		
	18.372	18.074	0.30	0.60	-0.60	0.66		
	17.946	16.875	1.07	1.37	-1.37	0.39		
	18.379	17.196	1.18	1.48	-1.48	0.36		
	17.900	16.880	1.02	1.32	-1.32	0.40		
	18.281	16.928	1.35	1.65	-1.65	0.32		
	18.127	16.720	1.41	1.71	-1.71	0.31		
	18.360	17.153	1.21	1.51	-1.51	0.35		
	17.248	16.881	0.37	0.67	-0.67	0.63		
