# Supplementary figures and images for: High-altitude Hypoxia Influences the Activities of the Drug-Metabolizing Enzyme CYP3A1 and the Pharmacokinetics of Four Cardiovascular System Drugs
Source: Pharmaceuticals (Basel). 2022 Oct 21;15(10):1303. doi: 10.3390/ph15101303 (PMC9612038; doi:10.3390/ph15101303)

The original pictures of western blotting as follows:

CYP3A1:

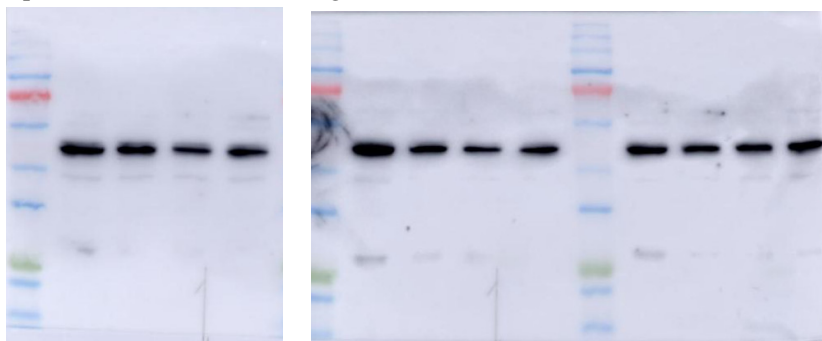

$\beta$ -actin:

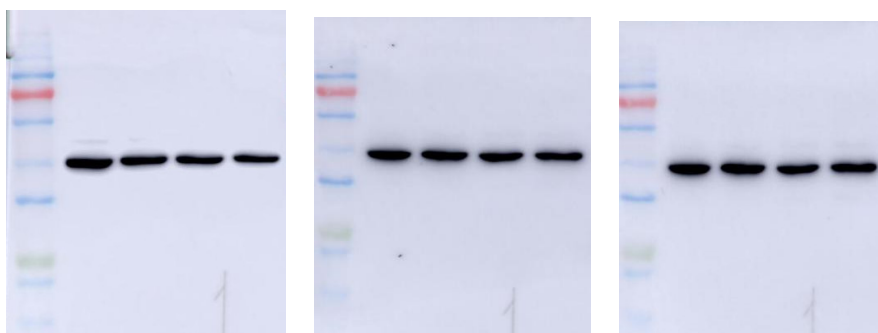

Supplement: Supplementary file 1 [file pharmaceuticals-15-01303-s001.zip › Figure S1.pdf]
